# Retraction statement: Dai, Y., Xie, F. and Chen, Y. (2020), Reduced levels of miR‐485‐5p in HPV‐infected cervical cancer promote cell proliferation and enhance invasion ability. FEBS Open Bio, 10: 1348–1361.

**DOI:** 10.1002/2211-5463.13622

**Published:** 2023-05-23

## Abstract

https://doi.org/10.1002/2211-5463.12869

The above article, published online on 28 April 2020 in Wiley Online Library (wileyonlinelibrary.com), has been retracted by agreement between the authors, the Editor‐in‐Chief, FEBS Press, and John Wiley and Sons Ltd. The retraction has been agreed following an investigation into concerns raised by a third party, which revealed inappropriate duplications between this and previously published articles [1–3]. Thus, the editors consider the conclusions of this manuscript substantially compromised.

Reference

1 Guan L, Ji D, Liang N, Li S and Sun B (2018) Up‐regulation of miR‐10b‐3p promotes the progression of hepatocellular carcinoma cells via targeting CMTM5. *J Cell Mol Med* **22**, 3434–3441.doi: 10.1111/jcmm.13620

2 Xu B, Xu T, Liu H, Min Q, Wang S and Song Q (2017) MiR‐490‐5p Suppresses Cell Proliferation and Invasion by Targeting BUB1 in Hepatocellular Carcinoma Cells. *Pharmacology* **100**, 269–282.doi: 10.1159/000477667

3 Butz H, Szabó PM, Khella HW *et al*. (2015 May) miRNA‐target network reveals miR‐124as a key miRNA contributing to clear cell renal cell carcinoma aggressive behaviour by targeting CAV1 and FLOT1. *Oncotarget* **6** (14), 12543–12557.doi: 10.18632/oncotarget.3815 PMID: 26002553; PMCID: PMC4494957

https://doi.org/10.1002/2211-5463.12869

The above article, published online on 28 April 2020 in Wiley Online Library (wileyonlinelibrary.com), has been retracted by agreement between the authors, the Editor‐in‐Chief, FEBS Press, and John Wiley and Sons Ltd. The retraction has been agreed following an investigation into concerns raised by a third party, which revealed inappropriate duplications between this and previously published articles [1–3]. Thus, the editors consider the conclusions of this manuscript substantially compromised.

**Reference**

1 Guan L, Ji D, Liang N, Li S and Sun B (2018) Up‐regulation of miR‐10b‐3p promotes the progression of hepatocellular carcinoma cells via targeting CMTM5. *J Cell Mol Med* **22**, 3434–3441.doi: 10.1111/jcmm.13620

2 Xu B, Xu T, Liu H, Min Q, Wang S and Song Q (2017) MiR‐490‐5p Suppresses Cell Proliferation and Invasion by Targeting BUB1 in Hepatocellular Carcinoma Cells. *Pharmacology* **100**, 269–282.doi: 10.1159/000477667

3 Butz H, Szabó PM, Khella HW *et al*. (2015 May) miRNA‐target network reveals miR‐124as a key miRNA contributing to clear cell renal cell carcinoma aggressive behaviour by targeting CAV1 and FLOT1. *Oncotarget* **6** (14), 12543–12557.doi: 10.18632/oncotarget.3815 PMID: 26002553; PMCID: PMC4494957

